# Tooth Pulp Afferents and Transient Receptor Potential (TRP) Ion Channels as Key Regulators of Pulp Homeostasis, Inflammation, and Pain

**DOI:** 10.3390/ijms27010182

**Published:** 2025-12-23

**Authors:** Man-Kyo Chung, Swarnalakshmi Raman, Arpad Szallasi

**Affiliations:** 1 Department of Neural and Pain Sciences, School of Dentistry, University of Maryland Baltimore, Baltimore, MD 21201, USA; sraman1@umaryland.edu; 2 UM Center to Advance Chronic Pain Research, University of Maryland Baltimore, Baltimore, MD 21201, USA; 3 Department of Pathology and Experimental Cancer Research, Semmelweis University, 1085 Budapest, Hungary

**Keywords:** transient receptor potential (TRP) channels, dental pain, tooth pulp, odontoblasts, pulpitis, TRPV1, TRPA1, pulp homeostasis

## Abstract

Dental pain often arises from the compromised integrity of the tooth pulp due to dental injury or caries. The dentin–pulp complex has long been considered to be central to the unique biology of dental pain. Most trigeminal ganglion afferents projecting into tooth pulp are myelinated neurons, which lose their myelination at the site of peripheral dentin innervation. The pulpal afferents likely combine multiple internal and external stimuli to mediate nociception and maintain pulp homeostasis. Transient receptor potential (TRP) ion channels in neurons and odontoblasts, along with mechanosensitive ion channels such as Piezo, form a key molecular hub for pulpal nociception by sensing thermal, chemical, and hydrodynamic stimuli. Among these, TRP vanilloid 1 (TRPV1) mediates nociception and the release of calcitonin-gene-related peptides (CGRPs), while TRP canonical 5 (TRPC5) mediates cold pain. TRP melastatin 8 (TRPM8) mediates the transduction of hyperosmotic stimuli. Pulpitis elevates endogenous TRPV1 and TRPA1 agonists, while inflammatory mediators sensitize TRP channels, amplifying pain. CGRP recruits immune cells and promotes bacterial clearance and reparative dentinogenesis, yet the roles of TRP channels in these processes remain unclear. Future studies should use advanced multi-omics and in vivo or organotypic models in animal and human teeth to define TRP channel contributions to pain, immune responses, and regeneration. Understanding neuronal and non-neuronal TRP channel interactions and their integration with other ion channels may enable novel analgesic and regenerative strategies in dentistry.

## 1. Introduction

Tooth pain is a defining symptom of many dental pathologies that significantly affect patient quality of life. Dental pain, commonly originating from the dental pulp, can vary from short-lived stimulus-evoked discomfort to chronic spontaneous pain. Globally, the overall prevalence of tooth pain in adults is 24% [1]. Persistent dental pain interferes with eating, sleeping, and daily functioning. Such pain is strongly associated with conditions such as pulpitis and dentinal hypersensitivity. The dentin–pulp complex is innervated by specialized trigeminal sensory afferents capable of detecting subtle mechanical, thermal, and chemical cues. The molecular and cellular mechanisms of intradental sensations are increasingly understood to involve not only neuronal architecture but also neuronal interactions with immune and non-neuronal cells within the pulp tissue. Among the sensory mediators, transient receptor potential (TRP) channels have emerged as pivotal in translating these stimuli into neural signals (for recent reviews, see [2,3,4,5]).

TRP channels, including transient receptor potential vanilloid 1 (TRPV1), TRP ankyrin 1 (TRPA1), TRP melastatin 8 (TRPM8), and TRPV4, are polymodal ion channels expressed on trigeminal neurons and pulpal cells [2,3,4,5]. These channels detect a range of stimuli such as temperature shifts, osmotic stress, mechanical deformation, and inflammatory mediators, and they participate in normal pulp physiology and pathological pain states. The relevance of TRP channels has been underscored in preclinical and clinical studies where they were found to modulate pulp inflammation, dentin sensitivity, and long-term changes in neural activity after injury or infection.

### 1.1. Scope of the Review: Peripheral Mechanisms Related to Pain Originating from Dental Pulp

Odontogenic pain arises from a variety of dental conditions involving tooth pulp (including dental caries, pulpitis, cracked teeth, and dentinal hypersensitivity) or the periodontal tissues (such as periapical periodontitis, acute periodontitis, and orthodontic pain). Periodontal pain is derived from distinct subsets of periodontal afferents and mechanisms [6]. In this review, we focus on pain mechanisms derived from the tooth pulp rather than periodontal tissues. Tooth pulp pain can be caused by reversible pulpitis (characterized by brief, stimulus-induced pain) or irreversible pulpitis, which presents as prolonged, spontaneous, or radiating pain [7]. The diversity of sensory neurons innervating the tooth likely contributes to the range of pain phenotypes observed under clinical settings. Aδ fibers innervating dentin and odontoblastic layers are responsible for the sharp pain induced by drilling and air blowing on exposed dentin or arising from the movement of dentinal fluid around the nerve terminals (the hydrodynamic theory; see Section 1.2 for details). In addition, this fiber group was shown to respond to cold temperatures [8], which suggests that cold receptors exist in dentinal Aδ fibers. C fibers are more likely to be present in the pulp proper rather than in the odontoblastic layer. It is likely that they do not respond to hydrodynamic stimuli but only to intense heat or cold that reaches the pulp proper [8,9,10], suggesting the expression of heat and cold-sensing molecules. The differential contribution of these two classes of fibers to thermal nociception in teeth has been proposed in human psychophysical (sensory perception) studies. In teeth, exposure to prolonged cold stimulation evokes biphasic cold pain; an immediate sharp pain followed by dull, radiating pain [8]. These responses can be matched with biphasic firing from dental afferents, which may be mediated by Aδ and C fibers, respectively, suggesting the expression of molecules responsible for temperature transduction in each fiber type. TRP channels may be implicated in the transduction of thermal and chemical noxious stimuli in these afferents, contributing to the maintenance of the sensitized state in pulpitis. Importantly, inflammation in the pulp may lead to ectopic or referred pain, reflecting alterations in the central processing of sensory input. Persistent orofacial pain following dental procedures may result from maladaptive neuroplasticity in central pain pathways, which involves central sensitization in the trigeminal sensory complex [11]. In this review, only peripheral mechanisms of transduction and modulations involving trigeminal afferents and tooth pulp tissues are discussed.

### 1.2. Mechanistic Hypotheses of Tooth Pain

**Figure 1:**
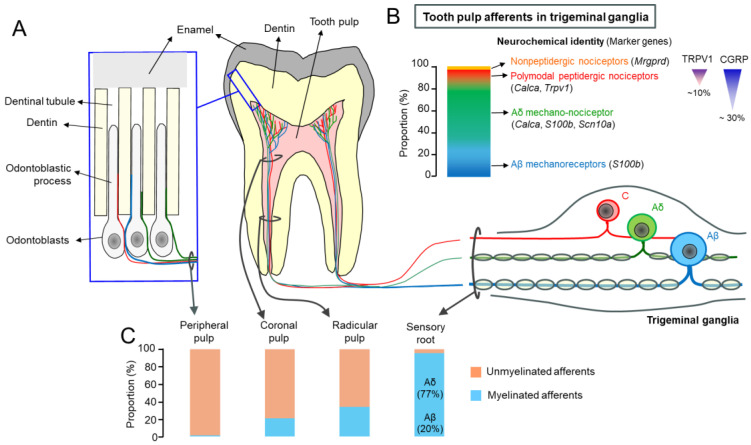
Tooth pulpal afferents (**A**) Diagram of a molar and odontoblasts. (**B**) Diagram of trigeminal afferents projected to tooth pulp and dentin, including major subtypes of pulpal afferents, marker genes, and their proportions in mouse trigeminal ganglia. (**C**) Proportion of myelinated and unmyelinated afferents in different sites along pulpal afferents. Reconstructed from Paik et al. [17] and Kim et al. [18]. Aβ afferents have a protective heavy myelin sheath that surrounds their axon, aiding in the fast conduction of action potential firing. In somatosensation, Aβ afferents contribute to tactile sensation [19]. Thinly myelinated Aδ afferents may conduct nociceptive inputs, leading to sharp pain sensation. Unmyelinated afferents lack this myelin sheath, leading to dull and lingering pain sensations. Peptidergic nociceptors mediate thermal and inflammatory pain, whereas non-peptidergic nociceptors are responsible for mechanical pain and injury-related nociception.

The tooth is composed of hard tissues, i.e., the outer enamel and inner dentin, that surround an inner loose connective tissue called dental pulp (Figure 1A). The pulp comprises a variety of cells, including fibroblasts, immune cells, blood vessels, and a layer of odontoblasts. The odontoblasts lining the inside surface of the tooth synthesize new dentinal tissue and add it to the inside of the tooth throughout life. Odontoblasts leave their long processes within the dentin, which results in the formation of dentinal tubules. Under healthy conditions, odontoblasts support secondary dentinogenesis, tissue integrity, and immune surveillance [12]. Upon noxious insults, such as caries or trauma, it responds by initiating tertiary or reparative dentin formation, recruiting immune cells, and activating stem cell–mediated regeneration [13]. Teeth are highly vascularized and innervated structures [14,15]. The pulpal afferents reach the root canal through the apical foramen where they join the neurovascular bundle [16]. In the coronal pulp, the sensory nerve endings form arborizing branches, known as the plexus of Raschkow. [This is a complex network of nerve fibers located in the dental pulp, just beneath the odontoblast layer. It was named after I. Raschkow who working in the laboratory of Purkinje provided the first description of this anatomical structure in his 1835 doctoral thesis, “*Meletemata circa mammalium dentium evolutionem*,” Breslau University.]

**Figure 2:**
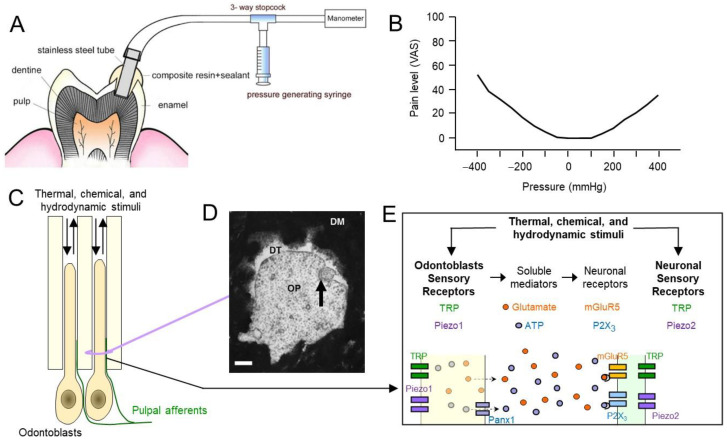
Potential mechanisms of dental pain mediated by hydrodynamic, neuronal, and odontoblastic-transduced mechanisms. (**A**) Experimental setup to determine pain levels by hydrostatic pressure on exposed dentin in human subjects. Pain level was assessed using the visual analogue scale (VAS; 0 no sensation to 100, the most severe pain). Reproduced from Charoenlarp et al. [30] with permission. (**B**) Both negative and positive pressures on dentin produce pain in humans. Modified from Charoenlarp et al. [30]. (**C**) Diagram of odontoblasts and sensory nerve terminals exposed to hydrodynamic stimuli. The arrows indicate movement of dentinal fluid. (**D**) Transmission electron microscopic image of a nerve terminal (arrow) juxtaposed with an odontoblastic process (OP) within the dentinal tubules (DTs) in human teeth. DM, dentin matrix. Scale bar, 200 nm. Reproduced from Carda and Peydro [20] with permission. (**E**) Diagram of the potential mechanisms of odontoblast-sensory nerve signaling. See text for references.

The sensory afferent terminals also terminate within the odontoblastic layers as well as within dentinal tubules [20,21]. In human teeth, the nerve terminals within dentinal tubules are juxtaposed to odontoblastic processes, and the intratubular axonal diameters are within the range of 136 nm to 636 nm (mean 336 nm) [20] (Figure 2). These afferents have cell bodies in the trigeminal ganglia (TG) whose central axons synapse with second-order neurons in the spinal trigeminal complex (Figure 1B,C). Pain is the predominant sensation regardless of the delivered stimuli, and, in healthy teeth, the intradental sensory apparatus is ‘silent,’ that is, it does not cause any perceivable sensation [22]. The odontoblasts, along with the nerve terminals projected into the odontoblastic layer, may modulate mechanical and chemical sensitivity within the tooth pulp, positioning the dentin–pulp interface as a dynamic sensory unit [2,3,4,5].

The dental nerves are mainly confined by the crown to the dental pulp, in a rigid chamber of dentin and enamel that protects the pulp from commensal oral microbial flora or drastic environmental changes within oral cavity. Once this protective chamber is compromised, dental hypersensitivity [23] or painful pulpitis can develop [24]. Indeed, dental caries is the leading cause that patients seek dental treatment [25]. Dental hypersensitivity is defined as pain arising in response to normally non-painful stimuli. The molecular mechanism of odontogenic pain (commonly called toothache) is only partially understood. The most widely held theories are as below, which have been reviewed previously in detail [2,26,27,28,29].

(1) The dentinal fluid hydrodynamic theory: external stimuli cause movement of the dentinal fluid that, in turn, excites the nociceptive afferents in the dental pulp. Both negative and positive pressure on dentin can induce pain in human subjects (Figure 2A,B) [30].
(2) The neuronal theory: external stimuli directly excite the nociceptive afferents by interacting with sensory transducing molecules, such as mechanosensitive Piezo2, temperature-sensitive TRP, and proton-activated acid sensing ion channels (ASIC) [31] (Figure 2C–E).
(3) The odontoblast transducer theory: odontoblasts act as sensory cells, transmitting noxious external stimuli to sensory nerve endings in the pulp. It was proposed that the ciliary organelle of the odontoblasts is the primary odontogenic nociceptor [31] (Figure 2C–E).

In this review, we discuss the roles of pulpal afferents and TRP channels in neuronal and non-neuronal cells to account for these hypotheses.

### 1.3. Rodent Models of Tooth Pain

Testing the above-mentioned hypotheses using in vivo models is challenging, and animal models used to determine the mechanisms underlying tooth pain are limited. The fact that tooth enamel, the hardest tissue in the body, surrounds the dental pulp precludes any application of pain-inducing materials to the pulp without disrupting the intact structure. Complete Freund’s adjuvant (CFA) is a mixture of mineral oils containing heat-inactivated *Mycobacterium tuberculosis* and is widely used to induce inflammatory pain in animal models. In dental research, CFA has been applied directly into the tooth pulp to study mechanisms of pulp pain [32,33]. The physical properties of enamel—high hardness and low thermal conductivity—also complicate the application of mechanical and thermal stimuli. Currently, several types of invasive and noninvasive methods are used to evoke pain in teeth. First, grinding the tooth surface to various degrees (with or without exposing the dental pulp) provides a model for evaluating the reaction of the dentin–pulp complex to pulp injury [34]. A second type of model uses both drilling of the tooth and the application of irritant chemicals, such as capsaicin, mustard oil, or bacterial lipopolysaccharide (LPS), to the cavity. This is done with exposure of the tooth pulp or with exposure of the dentin surface without exposure of the tooth pulp [35,36,37]. These models not only mimic the clinical features of irreversible pulpitis, such as sustained pain behavior and central sensitization, but also reveal the coordinated recruitment of neutrophils, macrophages, and T cells, along with the activation of cytokine-driven molecular networks that orchestrate pulp inflammation and repair processes—in which TRP channels are key modulators of nociceptive and inflammatory signaling [38,39]. Alternatively, non-invasive stimulation activates pulpal nociceptors without mechanical disruption of the tooth. Electrical stimulation of intact teeth [40] allows for the investigation of nociceptive signaling and molecular mechanisms while preserving tooth structure. Recent advancements in the genetic labeling of selected subpopulations of trigeminal afferents also allows for the activation of pulpal afferents through an optogenetic approach involving blue light exposure [41].

Quantifying dental pain in experimental animals is even more challenging. Due to the location of molars deep inside the oral cavity, assessing the responses to calibrated, direct stimulation of the teeth in rodents is not possible. Therefore, surrogate measurements of tooth pain have been used. For instance, the mechanical sensitivity of facial skin is used as a proxy for tooth pain [38]. This assumes that intense pulpal afferent inputs can drive molecular and cellular plasticity within the trigeminal ganglion. Such plasticity may cause ectopic pain or central sensitization, which then manifests as secondary hyperalgesia in facial skin [32,42,43]. In addition, spontaneous nocifensive behaviors, such as locomotion, exploration, or grooming-like behaviors, have been quantified following tooth injury in rats [44,45,46]. Changes in bite force, meal duration, or sucrose consumption after dental pulp injury have also been assessed [45,47,48]. Recent studies have also quantified spontaneous pain-like behaviors, including the mouse grimace scale, after tooth pulp stimulation [38,41]. Dental pulp injury has also been shown to increase anxiety-like behaviors in mice assessed using the elevated plus maze [38,49]. These approaches are instrumental in evaluating the contribution of specific molecular players such as TRP channels.

## 2. Tooth Pulp Afferents and Innervation

### 2.1. Pulpal Afferents in the Trigeminal Ganglia

The morphological features of nerves in dental pulp and dentin have been discussed in a recent review [50]. Briefly, the neurochemical identity of pulpal primary afferents has largely been studied in three different ways: first, by using retrograde chemical labeling of pulpal afferent neuronal cell bodies in the TG followed by the detection of marker gene expressions in intact ganglia through multiple approaches, such as immunohistochemical labeling or in situ hybridization; second, via retrograde chemical labeling of pulpal afferent neuronal cell bodies in the TG followed by enzymatic dissociation and single-cell based analysis of mRNA; and third, through immunohistochemical labeling or staining of nerve terminals within the tooth pulp.

Retrograde labeling of pulpal afferents in rodents allows for the investigation of their neurochemical properties by analyzing the labeled neuronal soma in histological sections of the TG, followed by immunohistochemical labeling of the protein of interest. The size range of pulpal afferents is mostly medium–large [17,51,52]. Pulpal afferents contain a subset of peptidergic neurons expressing calcitonin-gene-related peptide (CGRP). CGRP+ neurons make up approximately 30% of pulpal afferents in mice [53,54] and approximately 50% in rats [52,55]. The sizes of CGRP+ pulpal afferents are broadly distributed (small 31%, medium 43%, and large 26%) [53]. Non-peptidergic afferents binding to isolectin B4 (IB4) exist but are rare [53,56]. The sizes of Mas-related G-protein coupled receptor member D-positive (Mrgprd+) pulpal afferents are within the small to medium range. The neurochemical identities of pulpal afferents have also been investigated by analyzing the dissociated trigeminal neurons retrogradely labeled using DiI (a fluorescent dye) from the molars [57,58]. Although this allows a transcript analysis of isolated pulpal afferents via a highly sensitive methodology, some technical limitations (e.g., dissociation processes or mRNA amplifications) can skew the data. For example, the proportion of isolectin B4-positive (IB4+) pulpal afferents was estimated to be as high as 57% or 75% [57,58], which is inconsistent with other studies [53,56,59].

Recent studies using single-cell or single-nucleus sequencing methods have allowed for the clustering of TG neurons into distinct groups based on the unbiased assessment of marker gene enrichment [60,61] (Figure 1B). Taking advantage of the prominent markers in each cluster (C), in situ hybridization methods use the entire TG to simultaneously detect well-defined marker genes, which allowed for the clustering of trigeminal afferents into thirteen clusters [56]. This approach was adopted in combination with retrograde labeling from mouse molars to cluster pulpal afferents [54]. This study suggests that most of the molar pulpal afferents belong to myelinated neurons expressing *S100b* (87% to 96%). Between 59% to 76% of pulpal afferents expressed *S100b* (a Ca^2+^-binding protein) without *Calca*, the gene that encodes CGRP (C4 [56]), suggesting they are Aβ afferents (large-diameter, heavily myelinated, low-threshold mechanoreceptors that normally transmit non-painful touch and pressure). Between 20% to 28% of the pulpal afferents expressed *Calca* along with *S100b* (C6 [56]), suggesting they are Aδ nociceptors. Finally, between 4% and 13% of the pulpal afferents were composed of polymodal nociceptors expressing markers for polymodal nociceptors. While *Mrgprd*+ non-peptidergic pulpal afferents exist, they are rare. Interestingly, approximately 65% of S100b-expressing myelinated dental afferents expressed nociceptive neuron markers. *Scn10a* (encoding Nav1.8) and *Htr3a* (encoding the 5HT3 serotonin receptor) were expressed in most of the putative Aδ pulpal nociceptors and a subset of the putative Aβ afferents. A small subset of the putative Aδ pulpal nociceptors also expressed *Trpv1*, which accounted for approximately 5% of all the pulpal afferents. The mechanosensitive ion channel *Piezo2* was expressed in most of the pulpal afferents, including all *Trpv1*+ pulpal afferents.

Another recent study using RNA sequencing revealed the transcriptomic identity of pulpal afferents [59]. In this study, DiI was used to label maxillary molar afferents in mice, and the TG were dissociated to collect DiI-labeled single neurons. Then, mRNA from each neuron was reverse transcribed, amplified, and sequenced. The data were used to assign each neuron to one of the thirteen clusters mentioned above [56]. Among the 83 neurons, 64% were myelinated and 22% were putative Aβ neurons highly expressing *S100b* and *Ntrk3*, the gene encoding neurotrophic tyrosine kinase receptor type 3 (C4; 22%). Putative peptidergic Aδ mechanosensitive nociceptors expressing *S100b*, *Piezo2*, and *Calca* with a low expression of TRPV1 (C6) were the most abundant (42%). Polymodal peptidergic nociceptors (C7, C8, and C10) were also abundant (41%). Only one neuron was Mrgprd+ (C13) (a marker of mechanosensitive, nonpeptidergic afferents).

Another innovative approach further determined the neurochemical and functional properties of pulpal afferents [41]. Pulpal TG neurons were identified based on positive responses to electrical stimulation of the teeth by in vivo Ca^2+^ imaging. Then, in situ hybridization was performed to label key markers in the functionally identified pulpal afferents. In this study, 66% of the neurons expressed *S100b*, *Scn10a*, and *Calca*, whereas 23% expressed *S100b* only.

These findings indicate that pulpal afferents possess a distinctive neurochemical profile. The enrichment of nociceptive markers in *S100b*-positive myelinated fibers suggests that some Aβ-like pulpal afferents may contribute to pain signaling, reflecting an adaptation to the specialized environment of the pulp. This adaptation helps explain why tooth pain is intense, easily triggered by fluid shifts or pressure changes, and is rapidly exacerbated during inflammation. In addition, the presence of Aδ high-threshold mechanosensitive nociceptors, together with peptidergic CGRP-expressing fibers and polymodal nociceptors, underscores the molecular and functional diversity of pulpal innervation. The co-expression of mechanosensitive channels such as Piezo2 with nociceptive channels, including TRPV1, further supports a mechanistic link between mechanosensory transduction and nociception. Collectively, these studies support the idea that Aδ high-threshold mechano-nociceptors are enriched and likely play a major role in tooth pain.

### 2.2. Pulpal Afferent Terminals Within the Tooth Pulp

The axons of pulpal afferents form axonal bundles outside the TG. Light and ultrastructural microscopy studies performed on the proximal sensory root of the TG have established that parent axons innervating teeth are primarily composed of thinly myelinated Aδ fibers (76.8%), with a minor proportion of myelinated Aβ fibers (19.6%), and a small proportion of C fibers (4.4%) [17] (Figure 1C). As these axons pass the apical foramen and innervate the dentin, they lose the myelin sheath, such that the proportion of myelinated axons is only 34% in radicular pulp, 20.8% in coronal pulp, and only 1.2% in peripheral pulp [18]. Therefore, intradental nerves, especially in regions close to the dentin, are mainly unmyelinated axons originating from large-diameter neurons with myelinated axons. This change in myelination along the course of innervation partly explains the apparent discrepancy between the proportions of neurochemically distinct subsets of pulpal afferents between soma in the TG and nerve terminals within the tooth pulp. Another explanation is the extensive arborization of pulpal afferents. In rat molars, an arbor derived from a single axon from C pulpal afferents innervates as much as 80 µm × 350 µm of the dentinal surface [62]. Therefore, the density of neurochemically distinct subsets of unmyelinated nerve terminals near dentin is not proportional to the compositions of neurochemically distinct afferents in the TG, as discussed above.

Intradental nerves are mainly composed of several neurochemically distinct populations. First, parvalbumin (a marker of myelinated nerves)-expressing pulpal nerves are large-diameter axons (likely Aβ) that are extensively branched in the dentin and densely innervated [18,63]. Second, peripherin, an intermediate neurofilament, labels small to large diameter dental axons (likely C and Aδ) and is moderately branched to innervate dentin [64]. Peripherin+ intradental nerves do not overlap with parvalbumin [63]. Third, there is CGRP, a marker of peptidergic nerves and CGRP-expressing pulpal nerves, which are densely innervated into the dentin [64]. Paralvumin+ intradental nerves do not overlap with CGRP, and a minor subset of peripherin+ nerves are CGRP+ but not in terminals within the dentin [63]. In contrast, *Mrgprd* also labels a subset of dentin-innervating nerves, which completely overlap with peripherin [53,63]. A considerable proportion of parvalbumin+ and peptidergic pulpal afferents express vesicular glutamate transporter (VGLUT) 1 or 2, suggesting they are glutamatergic neurons in the molars of humans and rats [65]. Neonatal injection of capsaicin only modestly reduces unmyelinated pulpal afferent terminals, suggesting a minor composition of TRPV1+ pulpal afferents [66].

### 2.3. Functional Compositions of Pulpal Afferents

Despite the amount of accumulated knowledge regarding the identity of pulpal afferents, the functions of each subset are not well understood. This is mainly due to the lack of reliable methods to objectively assess tooth sensations in rodents. For example, pulpal afferents are enriched with myelinated A fibers. In electrophysiological recordings, this subset responds to the flow of dentinal fluid [67], suggesting its potential contribution to pain. Unfortunately, convincing evidence supporting the role of A pulpal afferents as nociceptors is lacking. A recent study has, however, shed light on this [41]. An in vivo Ca^2+^ imaging study showed that *S100b* and *Scn10a*-expressing intradental, high-threshold mechano-nociceptors respond to enamel damage. Surprisingly, approximately 50% of the intradental, high-threshold mechano-nociceptors responded to innocuous friction of the tooth surface, albeit in small amplitudes, suggesting that while one may not feel pain, sensory afferents still alert the brain to tooth damage. Importantly, chemogenetic activation of intradental *Scn10a*+ neurons in mice increased pain-like behaviors (i.e., hunching and grimacing). Furthermore, optogenetic activation of *Scn10a*+ neurons in intact mouse molars produced mandibular deflection. That study validated the idea that that intradental *Scn10a*+ neurons mediate behaviors related to pain and jaw reflex and offers a new conceptual framework for interrogating neurochemically distinct subsets of pulpal afferents in pain and tooth homeostasis.

## 3. Role of Neuronal TRP Channels in Dental Hypersensitivity and Pulpitis-Associated Pain

**Figure 3:**
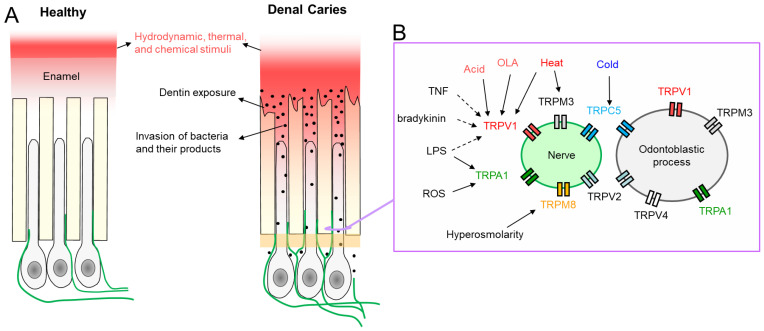
Thermosensitive TRP channels expressed in odontoblasts and tooth pulpal afferents (**A**) Pathological changes in teeth with dental caries. Thermal and chemical irritations are well limited to the superficial surfaces of teeth under healthy conditions. Dental caries destroys enamel and expose dentinal tubules so that odontoblastic processes and dentinal afferent terminals are more exposed to thermal and chemical irritants including bacterial products. (**B**) Expression of TRP channels and their potential roles in transducing various stimuli. See text for references. LPS, lipopolysaccharides; OLA, oxidized linoleic acid metabolites; ROS, reactive oxygen species. Dashed lines indicate sensitization or upregulation rather than direct activation.

The focus of this chapter is on the neuronal theory, with special emphasis on the emerging role that heat-responsive TRP channels, the so-called “thermoTRPs,” may play in the development and maintenance of temperature-driven dental pain [57]. The roles of TRP channels and their endogenous activation mechanisms in pulpal afferents are summarized in Figure 3.

Sensory afferents of the dental pulp have cell bodies in TG. As described above, individual molar teeth are served by a dedicated population of specialized TG neurons [54]. Notably, TG neurons that innervate the teeth and the cornea show distinct gene signatures [68]. For example, in dental primary sensory neurons *Cacna1b* (that encodes the pore-forming α-subunit of voltage-dependent Ca^2+^-channel, CaV2.2), *Trpv2*, and *Cnga4* (in olfactory sensory neurons, the product of this gene transforms chemical odorants into electrical signals by activating Ca^2+^-dependent chloride channels) showed the most robust expression. By contrast, corneal afferents were particularly rich in *Trpv1*. This is consistent with the common observation that chili pepper (capsaicin), the prototypical agonist of the TRPV1 receptor, is extremely painful when accidentally rubbed into the eye.

In the rat molar teeth, over 90% of the dental afferents are either thin myelinated Aδ [17] or unmyelinated C-fibers [66]. The central fibers of sensory TG neurons enter the brainstem where they form synapses with second order neurons in the spinal nucleus of the trigeminal nerve. Recently, the existence of an alternative efferent pathway was postulated in which the central TG fibers directly transmit nociceptive information into the brain [69]. The peripheral terminals of dental C-fibers are sites of release for pro-inflammatory neuropeptides, such as substance P and CGRP [70,71]. Combined, these neuropeptides initiate the biochemical cascade known as neurogenic inflammation. Neurogenic inflammation is thought to play a prominent role in the pathogenesis of pulpitis [71].

A distinct subset of C-fibers is identified by its unique sensitivity to capsaicin, the pungent ingredient in hot chili peppers (reviewed in [72,73,74]). The initial excitation of these nerves by capsaicin is followed by a lasting refractory state, traditionally termed “desensitization” [75], in which the previously excited neurons become unresponsive to a broad range of stimuli [74]. In adult rats, capsaicin desensitization is accompanied by characteristic ultrastructural changes in the affected neurons, most notably swollen mitochondria [76]. The molecular underpinnings of capsaicin desensitization are, however, only partially understood [77]. By definition, capsaicin desensitization is reversible [74]. By contrast, in newborn animals, systemic capsaicin administration selectively destroys a subpopulation of primary sensory neurons [78]. For example, in neonatal rats, capsaicin (50 mg/kg s.c.) was found to eliminate 64% of the unmyelinated fibers in the saphenous nerve [79]. Interestingly, the dental unmyelinated axons appear to be resistant to the neurotoxic action of neonatal capsaicin administration [66], which may be explained by low proportion of TRPV1-expressing dental afferents [54].

The receptor that recognizes capsaicin was cloned in 1997 as a heat-responsive channel [80]. Given the hot, burning sensation that capsaicin evokes in the oral mucosa, it was not really surprising that capsaicin activates a heat-responsive channel.

The capsaicin receptor belongs to the superfamily of TRP channels, named after a mutant channel in the eyes of fruit flies that conducts an abnormally brief (“transient”) Ca^2+^ response when exposed to continuous light [81]. The mammalian TRP channel superfamily has 28 members (27 in humans where TRPC2 is a pseudogene), divided into six subfamilies based on sequence homology: the canonical TRPC; the melastatin TRPM; the vanilloid TRPV; the mucolipin TRPML; the polycystin TRPP; and the ankyrin TRPA1 (reviewed in [82,83,84]). A recent cryo-electron microscopy study of the TRPV1 protein revealed fourfold symmetric channel with a central pore and large cytosolic domains [85]. The broad and diverse cellular expression pattern of various TRP channels implies diverse physiological functions. Defects in human genes encoding TRP channels (the so-called “TRP channelopathies”) are responsible for a number of hereditary disorders (reviewed in [84,86]). Within this group of disorders, human painful channelopathies represent an interesting subgroup [87]. For example, a gain-of-function mutation in the human *TRPA1* gene is responsible for Familial Episodic Pain Syndrome [88]. Rare *TRPA1* variants have also been found in a subset of patients with fibromyalgia or chronic widespread pain [89]. In the human cornea, a *TRPV1* gene “neuralgia mutation” (V527M) has been identified with enhanced proton-response [90]. Patients harboring this *TRPV1* variant are prone to develop persistent pain after LASIK surgery. Recently, TRP channels have emerged as major players in painful inflammatory conditions affecting the teeth, including pulpitis, periodontitis and dental erosion-induced pain [91]. It is currently unknown whether TRP gene variants play any role in painful dental conditions.

Within the TRP superfamily, the capsaicin receptor is the founding member of the vanilloid subfamily; hence its somewhat cumbersome name, TRPV1 (TRP superfamily, vanilloid subfamily, member-1). Vanilloid refers to the chemical moiety responsible for the pungency of capsaicin and its ultrapotent analog, resiniferatoxin [92]. In fact, the TRPV1 receptor was originally called “vanilloid receptor-1” (or briefly, VR1) [93].

TRPV1 is polymodal (reviewed in [94]): the main activators include capsaicin, noxious heat (over 42 °C) and changes in pH [80,95]. Phosphorylation of the TRPV1 protein by protein kinase C was shown to reduce the heat activation threshold [96]. Furthermore, protein kinase C can reverse the capsaicin-induced desensitization of TRPV1 [97]. TRPV1 is also a downstream target for well-known algesic compounds like bradykinin [98].

The Aδ fibers terminate at the pulp-dentin junction. Their activation is perceived as sharp stabbing pain (reviewed in [29]). External stimuli known to activate Aδ fibers include sweet, cold, and hypertonic agents. C-fibers are located deeper than Aδ fibers and they predominantly respond to heat, evoking the dull and aching pain associated with dental inflammation. Importantly, C-fibers are less sensitive to hypoxia than Aδ fibers, therefore during pulpitis they remain functional even when the thin-myelinated fibers are inactivated [99].

Since the first temperature-gated TRP channel, TRPV1, was cloned from rat sensory neurons [80], several other members of the TRP channel superfamily (TRPV2, TRPA1, TRPM3, TRPM8 and TRPC5) have been identified as putative molecular temperature sensors (reviewed in [100,101,102]. Combined, these channels cover a broad range of temperatures, from noxious hot to freezing cold. The thermodynamic and structural basis of temperature-dependent gating in TRP channels is beginning to be understood [103]. However, the exact role of these channels in physiological temperature sensation remains elusive [102]. For example, the “noxious heat sensor” TRPV1 (along with TRPM2) may sense warmth in ambient environment [104]. Warm sensation is, however, only blunted and not abolished in the TRPV1/TRPM2 double knockout mice [105]. Surprisingly, the inactivation of the cold-responsive TRPM8 was also required for the loss of ability to detect warm temperatures [105].

TRPA1 responds both to heat and cold, and now most authorities agree that this channel does not play any significant role in human physiological cold sensation (reviewed in [106]). Notably, TRPA1 displays an intrinsic bidirectional (U-shaped) thermosensitivity profile, which depends on the redox state of the receptor protein [107].

Apparently, there is a large degree in redundancy in noxious heat sensation: in the mouse, a “trifecta” of TRPV1, TRPA1 and TRPM3 deficiency is required for thermal insensitivity [108]. In men, the exact role of thermoTRPs in heat-sensation is less understood. In a clinical study with healthy volunteers, only TRPV1 contributed to the detection of noxious heat [109]. In accord, in clinical trials, TRPV1 (but not TRPA1 or TRPM3) antagonists were reported to elevate heat pain threshold, leading to minor burn injuries (reviewed in [110,111].

In afferents of rat maxillary molars, single-cell RT-PCR demonstrated the expression of three thermoTRPs, namely TRPV1, TRPA1 and TRPM8 [58]. Interestingly, some neurons seemed to express more than one thermoTRP. These afferents responded to both capsaicin and menthol, the prototypical agonists of TRPV1 and TRPM8, respectively [57]. Importantly, the same afferents were activated by both noxious heat (>43 °C) and cold (<17 °C) [57].

### 3.1. The Heat-Responsive Capsaicin Receptor, TRPV1

In rodents, neonatal administration of capsaicin is known to permanently eliminate sensory neurons that express the capsaicin receptor, TRPV1 [78]. In the incisor teeth of adult mice treated with capsaicin (50 mg/kg s.c.) as newborns, the number of C-fibers is 40% less than in controls [112]. Later studies, however, reported only minimal loss of pulpal C-fibers in rats subjected to neonatal capsaicin treatment [66,113]. Authors of the second study argued that dental C-fibers are unique in that they are remarkably resistant to the neurotoxic action of neonatal capsaicin administration [113]. Since both studies used electron microscopy to identify C-fibers, the cause of the discrepancy between these studies is unclear.

In patch-clamp experiments, the majority (65%) of adult rat dental sensory neurons identified by retrograde labelling responded to capsaicin [114]. In these cells, RT-PCR confirmed the presence of TRPV1 [114]. In immunohistochemical studies with retrogradely labeled TG pulpal afferents, the proportion of TRPV1 in pulpal afferents was as low as 10% [115,116]. Several lines of evidence indicate that pulpal TRPV1 is functional in animals. For example, in the canine teeth of cats with dentinal cavities, topical exposure to 1 µM capsaicin increases blood flow by 32% [117]. The capsaicin-evoked blood flow was no longer detected after denervation [118] or repeated capsaicin exposure, that is, desensitization [117]. In rats, exposure to heat (42 °C) also enhances pulpal blood flow in control, but not in capsaicin-desensitized animals [117]. In the rat maxillary tooth, capsaicin triggers neurogenic inflammation, evidenced by Evans blue extravasation [37]. Dental capsaicin responses were prevented by the TRPV1 receptor antagonist, capsazepine [114].

As discussed above, TRPV1-expressing sensory neurons are polymodal, that is they are activated by a number of unrelated stimuli, such as capsaicin, noxious heat and changes in pH. In accord, in the isolated superfused rat dental pulp, both heating and acidification were shown to enhance the capsaicin-evoked CGRP release. Conversely, cooling blocked the capsaicin effect [119].

In rat TG neurons identified by retrograde fluorescent dye applied to the upper molars, single-cell RT-PCR demonstrated TRPV1 mRNA [58]. Most of these neurons co-express CGRP as evidenced by the capsaicin-evoked CGRP release from pulpal afferents [119]. Intraganglionic resiniferatoxin (an ultrapotent capsaicin analog) administration ablates the Aδ and C-fibers, indicating that a subset of thin myelinated TG neurons also express TRPV1 [120,121]. This is interesting because in preclinical pain models inflammatory mediators were reported to induce TRPV1 expression in Aδ neurons that normally do not express this receptor [121]. If this observation holds true for pulpal afferents, *de novo* TRPV1 expression in thin myelinated axons may contribute to the acute pain that is experienced after molar extraction. Indeed, the TRPV1 receptor antagonist, AZD-1386, was reported to relieve pain after tooth extraction [122].

In rodents, the molecular signature of TRPV1-expressing sensory neurons (the “pain transcriptome”) indicates the existence of multiple sensory neuron subtypes [123]. Distinct “genetic fingerprints” were also reported in inherited sensory neuropathies [124]. Interestingly, the gene signatures of sensory TG neurons that innervate the mouse dental pulp, cranial meninges and cornea are distinct [68], paving the way for tissue-specific analgesia.

In rodents, TRPV1 expression in TG neurons is regulated by environmental factors. For example, both diabetic periodontitis [125] and LPS-induced pulpitis [115] were found to enhance TRPV1 expression in dental sensory neurons. Furthermore, in a rat model of pulp necrosis, sprouting of CGRP-positive sensory afferents was demonstrated [126]. By contrast, in dogs, fewer CGRP-positive afferents penetrate the dentine compared to rats, and the sprouting seen in the inflamed rat pulp is also absent [127]. This observation indicates marked species-related differences in dental C-fiber expression and warrants caution when extrapolating rodent observations to man.

TRPV1-like immunoreactivity was reported in healthy human tooth pulp afferents where it was significantly upregulated by caries [128]. Furthermore, TRPV1 expression was somewhat higher in painful caries compared to non-painful disease, although the difference in TRPV1 expression did not reach statistical significance [128]. Another study, however, found no difference in TRPV1 expression between intact and caries teeth removed from children [129]. Notably, this study used only immunofluorescence to detect TRPV1 and no functional assays. This is important because there is good evidence that TRPV1 in the human dental pulp is functional. In 16 of 20 healthy volunteers, capsaicin increased local blood flow in mandibular canine teeth [130]. Notably, the other 4 volunteers did not respond to capsaicin, indicating significant inter-individual differences in capsaicin sensitivity. In non-caries human wisdom teeth freshly extracted from 36 patients and sectioned to 1 mm slices, capsaicin stimulated CGRP release with considerable individual differences [131].

TRPV1 is clearly involved in the acute pain reaction that follows molar extraction. In rats, the “QX cocktail” (a combination of QX-314, a membrane non-permeable, voltage-sensitive Na^+^ channel blocker, and capsaicin that allows the neuronal entry of QX-314 via the open TRPV1 conductance) prevents the nociceptive behavior after tooth extraction [132]. The analgesic action of the “QX cocktail” (referred to as nociceptive anesthesia) was absent in TRPV1-null animals [133]. It was proposed that the “selective pain fiber nociceptive anesthetic strategy may provide an effective local anesthetic option for dental patients in the clinic” [133]. Prolonged corneal anesthesia achieved by topical QX-314 application in rats was also reported [134]. Notably, a recent study described TRPV1-independent neurotoxicity by QX-314 in human embryonic kidney (HEK)-293 cells transfected with the human TRPV1 isoform, raising concerns about the safety of the “QX cocktail” [135]. Currently, there are no published clinical trials with QX-314.

Women are more likely to experience orofacial pain than men [136]. In the teeth of healthy volunteers, the thermal pain threshold is lower in females, though this gender-related difference is no longer observed in patients with painful pulpitis [137]. The capsaicin-evoked CGRP release is about 50% higher in the incisor pulp of female rats compared to males [138]. Increased CGRP expression was reported in female rat TG neurons compared to males during CFA-induced inflammation [139]. In accord, capsaicin-evoked CGRP release from human dental pulp is maximal during the week prior to menstruation, and serotonin-induced increase in capsaicin-evoked CGRP release only occurs in women but not in men [70]. Combined, these observations imply an important role for female sex hormones in regulating capsaicin-evoked dental responses. In rats, both estradiol [140] and estrogens [141] were shown to exacerbate pain-related behavior via enhanced TRPV1 expression [141]. In humans, estrogens have also been implicated in painful conditions [142], including lower back pain and painful osteoarthritis [143].

Dental pain may also depend on age. In aged rats, experimental dental pain persists longer (over 7 h) than in young ones (disappears by 5 h) [144], despite the apparent loss of sensory afferents [145]. In aged rats, experimental dental pain is associated with higher tumor necrosis factor-α (TNFα) production [144].

The functional coupling between TRPV1 and TNFα is well-established. In human synovial cells, TNFα was shown to sensitize TRPV1 with resultant thermal hyperalgesia [146]. This may create a vicious cycle in the dental pulp in which heat activates TRPV1-expressing afferents, the activated afferents release sensory neuropeptides triggering neurogenic inflammation, and immune cells generate TNFα that further stimulate neuropeptide release by up-regulating TRPV1.

TRPM3 is another heat-activated channel [147], expressed in about half of TG neurons [148], including 20% of dental afferents. Co-expression of TRPM3 with TRPV1 and CGRP is common [148]. The role of TRPM3 in dental pain was proposed [31] but is yet to be investigated.

The expression of TRPV2 (formerly, VRL-1, a homologue receptor to TRPV1 with a higher noxious heat activation threshold of 52 °C [149]) was detected in 40% to 50% of dental TG neurons [150,151]. Unlike its close relative TRPV1 which is predominantly expressed in unmyelinated fibers, TRPV2 is present both in small C-type and large A-type sensory neurons [152]. In the tooth pulp, most (~95%) of the P2X_3_ receptor-positive afferents, which mediate rapid excitatory neurotransmission, co-express TRPV2 [153]. The function of TRPV2 in dental afferents is unknown. The TRPV2-null mouse displayed normal thermal and mechanical nociception [154]. In rat dorsal root ganglion (DRG), increased TRPV2 expression was noted during peripheral inflammation [155], and TRPV2 was implicated in the buccal mucosal pain that develops after surgical incision [156]. Thus, it is not unlikely that TRPV2 is involved in tooth extraction- and pulpitis-induced pain.

### 3.2. The Cold-Sensitive TRPM8 Channel

In a study of 461 healthy subjects, dental cold sensitivity was linked to *hTRPM8* gene single-nucleotide polymorphism [157]. Specifically, the rs12992084 variant of the *hTRPM8* gene was shown to confer increased cold sensitivity. [Parenthetically, the rs10166942 variant of the *hTRPM8* gene that causes reduced TRPM8 expression (and thus attenuated cold sensation) in humans is more prevalent in people living in cold climates [158]. This variant was also linked to reduced migraine risk [159]].

This was an exciting report since TRPM8 was cloned independently in two laboratories from rodent sensory neurons as a cold-sensitive channel [160,161]. [Notably, TRPM8 is also responsive to menthol, explaining the pleasant cooling effect of this natural compound.] Recently, a dedicated skin-to-brain cool sensation pathway was identified in mice that relays cool signals detected by TRPM8-expressing cutaneous nerve endings to the lateral parabrachial nucleus [162]. In volunteers, the small molecule TRPM8 antagonist, PF-05105679, was shown to ameliorate experimental cold pain induced by the cold pressor test [163].

TRPM8 mRNA was demonstrated by single-cell RT-PCR in a subset of dental primary afferents [58]. TRPM8-positive afferents are dense in the pulp and the dentinal tubules [164]. Most dental TRPM8-positive afferents co-express vesicular glutamate transporter-2 (VGLUT2), with increased expression during pulpitis [33,164]. These observations suggest that glutamate signaling by TRPM8-expressing pulpal afferents may be enhanced in the inflamed dental pulp, leading to inflammatory pain.

As expected, pulpal TRPM8 is activated by menthol [57]. Unexpectedly, TRPM8 was also activated by hyperosmotic sweet stimuli [165], implying a role in dental hypersensitivity. In fact, exposure to sweets is known to cause dental pain in patients with caries. Since TRPM8 is cold-responsive, it was a reasonable assumption that this channel also plays a role in cold-induced odontogenic pain. Yet, TRPM8 does not seem to contribute to cold sensitivity of the tooth pulp [166]. (Nor does the other cold-responsive TRP channel, TRPA1.) On the contrary, reduced axonal TRPM8 expression was reported in painful human teeth with cold hyperalgesia [167]. Notably, antisense knockdown of TRPM8 had no effect on cold hyperalgesia either in a rat model of chronic neuropathic pain [168].

Clearly, further studies are required to clarify the functional role (if any) of TRPM8 in dental cold sensation and cold sensitivity. It should be pointed out that more than half of cold-sensitive dorsal root ganglion (DRG) neurons do not express TRPM8, implying the existence of other (as yet unknown) molecular cold sensors. In keeping with this, TRPM8-null mice avoid contact with surfaces that are below 10 °C [169].

### 3.3. Other thermoTrps

TRPA1 is the only TRP channel that is specialized for sensing chemically reactive compounds generated during disease, for example, methylglyoxal in painful diabetic neuropathy [170,171], as well as harmful substances like diesel exhaust [172] or tear gas [173] in external environment. The role of TRPA1 in physiological temperature sensation is species-dependent in animals, and is controversial in humans (reviewed in [174]). In clinical studies, TRPA1 antagonists did not compromise heat or cold sensation [175].

Unexpectedly, individuals carrying a homozygous loss-of-function *TRPV1* gene mutation (N331K) showed increased sensitivity to noxious cold [176]. The same individuals also exhibited increased neurogenic inflammatory, flare and pain reactions in response to TRPA1 agonists [176]. These observations suggest negative functional control of TRPA1 by TRPV1. There is good experimental support for this model. Functional TRPV1/TRPA1 heteromers were already demonstrated [177]. In DRG neurons, the TRPV1/TRPA1 heteromers form a complex with Tmem-100 [178]. Tmem-100 weakens the association between TRPV1 and TRPA1, thereby liberating TRPA1 from the inhibitory control of TRPV1. (Tmem-100 (Transmembrane protein-100) also “awakens” silent nociceptors during inflammation [179].)

TRPA1 expression was demonstrated in a small subset of dental primary afferents [58]. In dental afferents, the expression level of TRPA1 was low and did not correlate with cold sensation [166]. One study found increased TRPA1, but not TRPV1, expression in teeth with caries [129]. Moreover, *Porphyromonas gingivalis* LPS was reported to activate sensory TG neurons, triggering CGRP release [180]. Taken together, these observations imply that during pulpitis neuronal TRPA1 may play a role in the neurogenic inflammatory reaction.

Bleaching (whitening) by peroxide is a popular means to improve the appearance of discolored teeth. However, many dental patients find bleaching an unpleasant experience. Peroxide is known to stimulate TRPA1 [181] via a reversible thiol-modification reaction [182]. These observations may explain why bleaching hurts [183]. Bleaching agents can penetrate enamel, especially in teeth with enamel crack [184], and alter dentinal structure and protease activities in dentin and pulp tissues [185]. Incisors have relatively thin dentin. Hydrogen peroxide and its free-radical byproducts can exert cytotoxic effects on odontoblasts, leading to cellular stress, inflammatory signaling, and potential pulpal irritation or damage. The extent of hypersensitivity also depends on dentin thickness, permeability of the dentinal tubules, and the degree to which peroxide can diffuse toward the pulp. These structural and biological variables likely interact with peripheral nociceptor activation to produce the overall pain response [186].

Notably, recently TRPA1 expressed in TG neurons has been implicated in the development of orofacial neuropathic pain [187]. In this study, the mechanical allodynia that occurred in the whisker pad of mice following infraorbital nerve injury was dependent on the generation of fibroblast-derived interleukin-33 (IL-33). In turn, IL-33 facilitated the membrane expression of TRPA1. Fibroblast ablation in TG resulted in decreased IL-33 production and attenuated pain behavior. Mechanical allodynia in response to intraganglionic human recombinant IL-33 injection was abrogated by silencing of the *Trpa1* gene. Taken together, these observations imply that fibroblast-derived IL-33 facilitates the excitability of TG sensory neurons (and resultant neuropathic pain) by upregulating TRPA1 expression [187]. Another study, however, linked the neuropathic pain caused by infraorbital nerve injury to insulin-like growth factor-1 (IGF-1) produced by macrophages infiltrating the injured nerve [188]. IGF-1 upregulated the expression of TRPV2 and TRPV4 in sensory neurons that innervate the whisker pad. Since both TRPA1 [189] and TRPV4 [190] are candidate mechanosensitive channels, it is plausible that the IL-33/TRPA1 [187] and IGF-1/TRPV4 [188] pathways play a complementary role in the development of nerve injury-induced mechanical allodynia. The involvement of these pathways in pain after tooth injury needs to be determined in the future.

**Figure 4:**
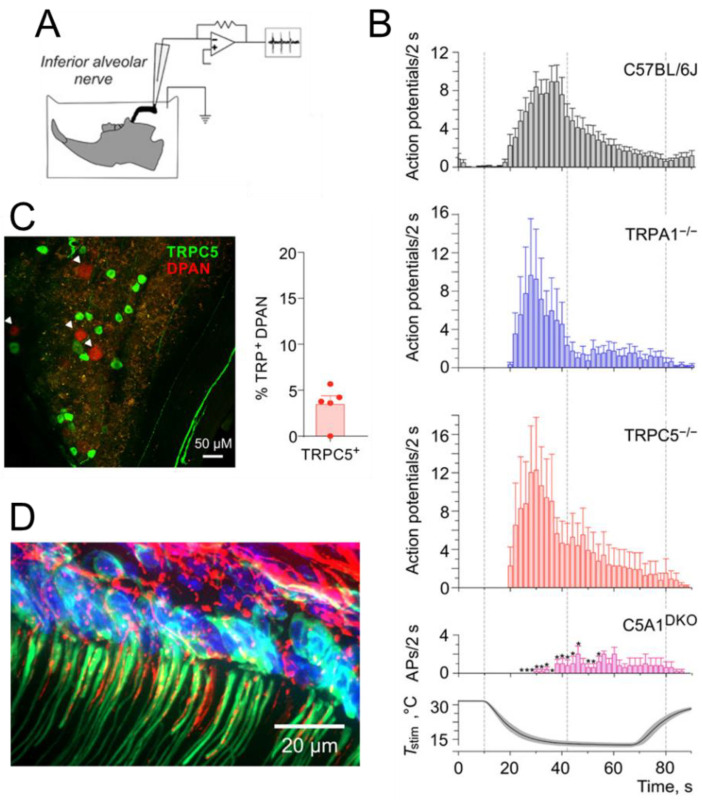
TRPC5 transduces cold temperature in mouse tooth pulp. (**A**) Diagram of jaw nerve preparation for ex vivo electrophysiological recordings of cold responses. (**B**) Histograms of action potentials during cold stimulation in jaw nerve preparation from C57BL/6J, TRPA1 knockout, TRPC5 knockout, and TRPA1/TRPC5 double knockout (C5A1DKO). * *p* < 0.002 vs. C57BL/6J in Student’s *t*-test. (**C**) Approximately 4% of dental pulp afferent neurons (DPAN) in trigeminal ganglia are TRPC5+. DPAN were retrogradely labeled from molars using carbocyanine dye. TRPC5 was labeled using immunohistochemistry. (**D**) TRPC5 are expressed in odontoblasts of mouse molar. Immunohistochemical labeling of green fluorescent protein (GFP; green) and βIII-tubulin (red), a neuronal marker. Blue, DAPI. The figure is reproduced from Bernal et al., 2021 [192].

TRPC5 was identified as a cold transducer in the peripheral nervous system [191]. As discussed below, odontoblast TRPC5 is thought to play a role in tooth cold sensitivity [192]. TRPC5 expression in dental primary afferents is also reported [192]. According to a recent study, approximately 75% of human sensory neurons express TRPC5 [193]. Therefore, the presence of TRPC5 in dental sensory afferents would not be unexpected. Electrophysiological responses to cold stimuli in jaw nerve preparations are greatly reduced in mice with double knockout of TRPA1 and TRPC5, suggesting their concerted contributions in tooth cold sensitivity [192] (Figure 4).

### 3.4. TRP Channel Expression in Pulpitis

Pulpitis is the inflammation of the dental pulp due to trauma or infection. Unfortunately, animal models of human pulpitis are still lacking. One problem is that the histopathologic changes and pain symptoms in human pulpitis are poorly correlated.

Clinically, pulpitis has been divided into symptomatic (painful) and non-symptomatic (non-painful) forms. The principal cause of pulpitis is untreated tooth decay. The pathogenesis of caries is beyond the scope of this review. Briefly, fermentable carbohydrates produced by commensal oral bacteria may produce a cariogenic niche that, if left untreated, can progress into tooth cavity, exposing the dental pulp environmental stimuli [194] (Figure 3A). LPS, a bacterial toxin, was reported to activate cultures TG neurons and sensitize TRPV1 channels by a toll-like receptor 4 (TLR4)-mediated fashion [195]. Notably, LPS was also found to up-regulate TRPA1 independent of the TLR4 pathway [180,196]. In a rodent model of acute pulpitis, consistent up-regulation of TLR4 was observed in TG neurons, ipsilateral to the injured pulp [197]. Retrograde labeling identified the neurons with up-regulated TLR4 expression as small to medium size cells, co-expressing TRPV1 and CGRP [197]. Blocking the TLR4 signaling in TG neurons reduced the nociceptive behaviors [197]. In a rat model of acute pulpitis induced by pulp exposure to CFA, increased TRPV1 expression was found in the pulpal afferents [198]. Interestingly, the application of CFA to the first molar increased the expression of TRPV1 in TG afferents retrogradely labeled from the adjacent second molar [32] or from facial skin. The administration of a TRPV1 antagonist into facial skin reduces mechanical hyperalgesia in facial skin following pulpal inflammation [42], suggesting that ectopic upregulation of TRPV1 in TG can contribute to the ectopic pain under pulpitis conditions.

A key molecular event of pulpitis is increased microcirculation. Sensory neuropeptides (most notably, substance P and CGRP) released from TRPV1-expressing afferents augment pulpal blood flow and triggers neurogenic inflammation [199]. CGRP contributes to recruitment of neutrophils and monocytes after pulp injury, which in turn increases spontaneous pain-like behaviors in mice [200]. Moreover, many inflammatory cells have receptors for substance P and produce pro-inflammatory cytokines, including interleukins and TNFα. As discussed above, TNFα can sensitize TRPV1 [146,201], creating a vicious cycle. Notably, marked increase in substance P was reported in human caries [202] and symptomatic irreversible pulpitis [203,204,205]. The expression of the substance P receptor NK1, is also elevated in human pulp tissue samples collected from caries [206]. One may argue that bacterial LPS stimulates substance P production by generating TNFα [207]; in turn, substance P causes dental pain. In fact, in a randomized clinical trial, a positive correlation was found between substance P levels and postoperative endodontic pain in patients with irreversible pulpitis [208]. However, causal contributions of substance P to pulpitis in animal models is not established yet, although its role in periodontitis is known [209].

Bradykinin is a known upstream activator of TRPV1 [210], further cementing the link between pulpitis-associated pain and TRPV1. Bradykinin evokes dull dental pain in healthy volunteers, presumably by activating C-fibers [28]. Bradykinin levels are significantly elevated during irreversible pulpitis [211], contributing to inflammation and pain.

Bacterial LPS, TNFα, and bradykinin sensitize and/or stimulate TRPV1 indirectly. The existence of endogenous TRPV1 agonists (so-called endovanilloids) was also postulated. It is believed that these substances are generated on demand during inflammation. For example, the inflammatory soup is usually acidic and protons are known direct activators of TRPV1 [95,212].

Oxidized linoleic acid metabolites were shown to directly activate TRPV1 in animal models of post-burn pain [213] and inflammatory pain [214]. Oxidized linoleic metabolites may also function as endogenous TRPV1 activators in the inflamed human dental pulp [215]. These substances are enzymatically generated by cytochrome P450, and the cytochrome P450 inhibitor, ketoconazole, inhibits experimental inflammatory pain [216]. Cytochrome P450 expression and resultant linoleic acid metabolism are increased in pulpitis compared to healthy dental pulp [215]. Tissue extract obtained from inflamed human dental pulp elicits a capsaicin-like inward current in TG neurons which is inhibited by the TRPV1 receptor antagonist, iodo-resiniferatoxin [217].

## 4. Roles of TRP Channels in Odontoblasts in Tooth Pain

### 4.1. The TRPV Subfamily

For a long time, capsaicin was considered to be selective for a specific subset of primary sensory neurons (reviewed in [72,73]). In fact, capsaicin was widely used by pharmacologists as a tool to dissect these neurons and study their contribution to physiological functions and disease. Although non-neuronal capsaicin actions were reported, most authorities considered these actions non-specific, that is not mediated by the capsaicin receptor (see [74]).

Following the molecular cloning on the capsaicin receptor TRPV1 [80], an antisense RNA probe containing nucleotides 410 to 12,015 of the rat *Trpv1* gene was used in a solution hybridization/nuclease protection assay to explore the tissue distribution of TRPV1 [218]. In rats, other than in DRG, robust TRPV1 expression was detected in a few brain areas (such as the cerebral cortex and the hippocampus), and a much weaker signal was detected in kidney, spleen and skeletal muscle [218]. In human tissue, a similar approach detected a strong signal in kidney and a very weak signal in temporal cortex and cerebellum [219].

Subsequently, confusingly widespread TRPV1 expression was reported in practically every human cell and tissue. Some of these studies employed anti-TRPV1 antibodies that later turned out to be non-selective for TRPV1, or antagonists that were eventually recognized to inhibit several targets other than TRPV1. Therefore, reports of unexpected TRPV1 expression have to be carefully re-examined.

Recently, TRPV1 mRNA expression in mouse and human tissues has been determined by sensitive RNA-sequencing (reviewed in Koivisto et al. [111]). As expected, sensory ganglia (DRG and TG) exhibited the highest expression. In humans, TRPV1 was also reliably detected in skin keratinocytes. The level of TRPV1 expression in other tissues was quite low which is likely to result in inconsequential amounts of TRPV1 channel protein synthesis ([111]). Notably, human keratinocytes also express a dominant negative form of TRPV1 (TRPV1b), which confers functional resistance to capsaicin in human keratinocytes [220].

With these sobering thoughts in mind, let’s review the evidence of TRPV1 expression in odontoblasts. In patch-clamped odontoblasts isolated from the incisor teeth of newborn rats, capsaicin-induced inward current was reported that was inhibited by the first-generation capsazepine [221]. In primary rat odontoblasts in culture, RT-PCR detected TRPV1 mRNA and fluorometric Ca^2+^ imaging demonstrated heat-induced currents [222]. A different rat study, however, was unable to confirm these findings: no TRPV1 was detected in odontoblasts by immunohistochemistry or single-cell PCR, and neither capsaicin nor heat activated these cells [223].

In human odontoblasts isolated from healthy (non-caries) wisdom teeth, functional TRPV1 expression was reported by a combination of immunohistochemistry, RT-PCR, and functional assays such as inward Ca^2+^ currents in response to capsaicin or heat [224]. Somewhat unexpectedly, odontoblast TRPV1 was activated by stretching the cell membrane [225]. This study implies a potential role for TRPV1 in sensing hyperosmotic stimuli such as sweets. Indeed, in human odontoblast-like cells, capsazepine blocks TRPV1 activation by hyperosmotic stimuli (also protons and heat) [226]. In human odontoblast-like cells, TRPV1 is present in the membrane, mitochondria and endoplasmic reticulum [227].

In summary, the gamut of experimental evidence points to functional TRPV1 expression in human odontoblasts. It was speculated that odontoblast may function as sensory receptor cells [221], akin to TRPV1-expressing keratinocytes and urothelial cells. [Notably, a different group could not detect TRPV1 expression in human odontoblast-like cells [228]].

In addition to TRPV1, in acutely isolated odontoblasts TRPV2 [225,227], TRPV3 [227] and TRPV4 [225,229] expression was reported by various groups. Hypotonic solution-induced membrane stretching of plasma membrane activates mouse odontoblast, implicating the stretch- and osmolarity-sensitive TRPV2 and TRPV4 in this effect [225]. In keratinocytes, TRPV3 is warm-sensitive [230] and it may play a similar role in the teeth.

### 4.2. The TRPM Subfamily

Three members of the TRPM subfamily have been reported in rodent odontoblast: TRPM3 [222], TRPM7 [231] and TRPM8 [224,232]. Notably, these reports are in conflict with each other since the TRPM3 report failed to detect TRPM8 [222], whereas the TRPM7 study failed to detect TRM3 in odontoblasts [231]. The expression of TRPM8 in freshly isolated human odontoblasts was also described by a combination of RT-PCR and immunohistochemistry [233].

In DRG neurons, TRPM3 is a noxious heat-sensitive channel [147], expressed in a large subset of somatosensory neurons where it mediates responses to the endogenous neurosteroid, pregnenolone sulfate. [Parenthetically, TRPM3 also detects pregnenole sulfate in pancreatic β-cells [234] and vascular smooth muscle [235]]. TRPM3-deficient mice fail to develop inflammatory thermal hyperalgesia [147]. Conversely, increased TRPM3 expression was observed in sensory neurons that innervate inflamed tissues [236]. This functional TRPM3 upregulation plays a prominent role in acute pancreatitis pain [237] and cyclophosphamide-induced cystitis pain [238]. Conversely, TRPM3 null mice were protected from cold and mechanical hypersensitivity in the oxaloplatin model of chemotherapy-induced neuropathic pain [239].

As evidenced by patent literature, there is considerable interest in developing potent and selective TRPM3 antagonists as novel analgesic agents (reviewed in [240]). In fact, one TRPM3 antagonist, BHV-2100 (Biohaven Pharma, New Haven, CT, USA), is about to enter clinical trials (https://clinicaltrials.gov/study/NCT06603623; accessed on 6 October 2025).

TRPM7 is expressed in the majority (87%) of rat odontoblasts [231]. TRPM7 is tightly regulated by Mg^2+^. In accord, TRPM7 is believed to play an important role in dentine mineralization by regulating Mg^2+^ uptake [231]. TRPM7 is also expressed in ameloblasts. The conditional TRPM7 knockout mouse exhibits impaired enamel calcification [241], confirming the crucial role of TRPM7 kinase domain in the early stage of amelogenesis [242]. In rat odontoblasts, TRPM7 may also function as a mechanoreceptor [243].

Similar to sensory neurons, TRPM8 expressed in odontoblasts responds with Ca^2+^ influx to menthol and temperature changes (<22 °C) [224,232].

### 4.3. TRPA1

The expression and function of TRPA1 in odontoblasts is confusing. Several groups reported functional TRPA1 expression in acutely isolated rat and human odontoblasts [224,228,232,244,245,246]. Others, however, failed to detect any TRPA1 mRNA and/or protein [222,223,233].

As mentioned above, TRPA1 is the only TRP channel that recognizes chemically reactive compounds [247]. TRPA1 is also activated by pungent spice ingredients, like allicin in garlic and allyl isothiocyanate in wasabi [247]. Moreover, in rat odontoblasts TRPA1 may function as a mechanoreceptor [232]. If TRPA1 is expressed in odontoblasts (which remains questionable), this channel may contribute to pain in response to chemical substances generated during pulpitis.

### 4.4. TRPC5, a New Kid on the Block?

TRPC4/C5 is thought to regulate negative pain-related emotions, such as fear and anxiety, in the brain [248]. The pain phenotype of the TRPC5 knockout mouse is, however, confusing. One group reported reduced mechanical allodynia in various pain models [193], whereas a different group described enhanced pain-related behavior during experimental arthritis [249]. Albeit at low levels, TRPC5 is also expressed in sensory neurons [250], as well as in non-neural cells, including odontoblasts [192]. In healthy teeth, TRPC5 may function as a physiological cold sensor [192]. Indeed, human TRPC5 exhibits intrinsic cold sensitivity that depends on the phosphorylation state of the receptor protein [251]. One may argue that TRPC5 may also be responsible for the cold-evoked dental pain experienced by patients with tooth decay. TRPC5 is upregulated in pulpal afferents in human molar with dental caries [192].

## 5. Non-TRP Channel Mechanisms in Dental Afferents and Odontoblasts

### 5.1. Non-TRP Channels in Pulpal Afferents

The distinct mechanosensory and nociceptive characteristics of tooth pulp suggest that, in addition to TRP channels, a number of non-TRP ion channels are expressed in dental primary afferents. In this section, we briefly discuss the potential roles of non-TRP channels in pulpal afferents under pathophysiological conditions.

Dental tissues innervated by trigeminal sensory neurons contain Piezo2, a mechanosensitive ion channel necessary for tactile sensation and mechano-nociception [252]. Piezo2 expression and deactivating mechanosensitive currents have been found in dental primary afferent neurons, many of which also co-express Nav1.8, CGRP, and NF200 [253]. Pharmacological blockers or small interfering RNA knockdowns eliminate these Piezo2-mediated currents, demonstrating their function in mechanotransduction. Piezo2 is likely a crucial mediator of dental mechanotransduction. Piezo2 can participate in pathological hypersensitivity because teeth with dentinal sensitivity are responsive to mechanical perturbations such as shifts in dentinal fluid. By reducing the activation threshold of sensory afferents, Piezo2 activity may be a factor in mechanical hyperalgesia in inflamed dental pulp [252,253]. However, a recent study found that pulpal afferent responses to tooth damage were not dependent on Piezo2 [41]. Further studies are needed to decipher Piezo2-mediated mechanosensitivity and mechanical nociception in pulpal afferents.

In certain nociceptors, TACAN, an ion channel encoded by *TMEM120A*, may act as a mechanosensitive protein. Its expression pattern suggests that it might play a role in pulpal mechanotransduction, even though its precise channel properties remain debatable. By maintaining afferent excitability, TACAN may work in tandem with Piezo2 to encode mechanical forces within the pulp and could be a factor in chronic pain states [254,255].

Acid-sensing ion channels (ASICs) are proton-gated cation channels that are activated under acidic conditions. ASIC3, which is especially expressed in trigeminal nociceptors, plays a role in both mechanical and inflammatory pain. Approximately 1/3 of pulpal afferents express ASIC3, some of which are colocalized with CGRP [256]. The acidic extracellular milieu in pulpitis and caries progression may activate ASIC3, causing nociceptor depolarization and increased pain sensitivity. Thus, ASIC3 is a crucial molecular mediator that connects pulpal pain and local acidosis [257].

Nociceptive afferent excitability is regulated by voltage-gated sodium channels, with Nav1.7 playing a particularly important role in dental pain mechanisms. Human pain syndromes are strongly linked to Nav1.7, which is highly enriched in trigeminal neurons. Nav1.7 in pulpal afferents probably increases nociceptive transmission in inflammatory conditions by facilitating ectopic discharges and lowering the threshold for action potential initiation [46,258].

Odontoblasts, immune cells, and damaged pulp tissue release extracellular ATP, which acts as a strong excitatory signal for sensory afferents. P2X_3_ and P2X_2/3_ receptors are highly expressed in trigeminal afferents [259,260,261,262]. Purinergic signaling may play a crucial role in odontoblast-afferent communication, whereby the release of ATP triggers sensory fiber activation and pain perception. P2Y receptors influence neuronal excitability and play a role in pulp neuroinflammatory signaling. These purinergic systems work together to offer a vital link between nociceptive transmission and pulp tissue homeostasis [263,264,265,266].

### 5.2. Potential Mechanisms of Communication Between Odontoblasts and Pulpal Afferents

Sensory signaling often involves communication between non-neuronal cells that detect environmental changes, with adjacent sensory terminals transmitting the signals to the brain. For example, taste signaling involves taste buds and gustatory nerve interactions facilitated through ATP [267], while tactile sensation involves serotonergic signaling between Merkel cells and Aβ fibers [268]. The activation of skin keratinocytes is sufficient to evoke pain-like behaviors in mice [269,270]; likely through direct contacts or chemical mediators such as prostaglandins and ATP [271,272,273]. TRP channels are essential molecular sensors in odontoblasts and tooth pulp afferents, and there is a growing body of work suggesting the existence of odontoblast-nerve communication pathways [3,274,275] (Figure 2E). These pathways, including glutamatergic and purinergic transmissions, likely function in coordination with (or downstream of) TRP activation, providing a more comprehensive framework for understanding inflammation, pain, and pulp homeostasis.

Odontoblasts are capable of glutamate signaling. Rat odontoblasts contain glutamate and express TRPV1, TRPA1, and TREK1 (a two-pore-domain K^+^ channel), while nearby pulpal afferents express the metabotropic glutamate receptor mGluR5 [244]. Following dentin damage, glutamate levels in odontoblasts and axonal mGluR5 rise, and glutamate is released by cultured odontoblasts in a Ca^2+^-dependent manner [244]. Furthermore, mechanical stimulation of individual odontoblasts causes glutamate release through glutamate-permeable anion channels, which, in turn, activates mGluRs in nearby odontoblasts and co-cultured trigeminal neurons [276]. Therefore, odontoblasts can act as non-neuronal sensory detectors that translate environmental cues into chemical signals. By facilitating the Ca^2+^ entry necessary for glutamate release, TRPV1/TRPA1 activity may connect TRP activation to glutamatergic transmission downstream.

Another mechanism of odontoblast-afferent crosstalk is ATP signaling. The activation of TRP channels induces ATP release from odontoblasts to activate adjacent odontoblasts and sensory afferents [228,274,275]. Odontoblasts have strong ATP-evoked Ca^2+^ responses and express several purinergic receptors (P2Y_2_, P2Y_4_, P2X_2_, P2X_4_, P2X_6_, and P2X_7_) [277]. Trigeminal neurons are activated by odontoblasts’ release of ATP, which also leads to inflammatory hyperalgesia [264,278,279]. As with skin keratinocytes—where ATP and cytokine release modify cutaneous sensory fibers, and taste buds, where ATP is the primary transmitter to gustatory afferents—ATP may be a crucial non-TRP mediator in pulp sensory transmission. Moreover, TRPV channel activation may directly promote ATP release by raising intracellular Ca^2+^, which would enhance nociceptive signaling.

A further dimension is provided by mechanosensory pathways. Piezo1 and Piezo2 were reported to be expressed in odontoblasts, suggesting their joint role in mechanical sensitivity [280,281,282]. In response to changes in dentinal fluid, odontoblasts may activate mechanosensitive mechanisms, e.g., likely through Piezo1 and Piezo2, that may induce the release of glutamate or ATP without the assistance of TRPs. This is similar to how skin keratinocytes can transform mechanical stress into paracrine signaling that makes afferents more sensitive and how Piezo-mediated mechanotransduction occurs in sensory systems [283]. In rodents, pharmacological inhibition of Piezo1, Pannexin 1, or P2X3 reduces nociception-like behaviors evoked by cold water stimulation on exposed dentin [281]. Close spatial localization of odontoblasts and P2X_3_-expressing neurofilament-H-positive nerves (likely Aδ fibers) suggests that Piezo1 activation in odontoblasts can trigger ATP release through pannexin-1 hemichannels, which, in turn, activates P2X3 in nerve terminals [281]. The role of odontoblasts in dentinal sensation was further determined by ablating odontoblasts using diphtheria toxin receptor (DTR) expression driven by *Col1a1*-Cre. In this system, cells expressing DTR become selectively susceptible to diphtheria toxin, which inhibits protein synthesis and leads to cell death. A week of diphtheria toxin administration ablated odontoblasts, and pain-like behaviors after cold water stimulation were significantly reduced despite intact P2X_3_^+^ fibers. These findings suggest that the Piezo1-Pannexin 1-P2X_3_ signaling axis translates dentinal stimuli into the excitation of neurons, likely establishing odontoblasts as mechanosensory bridges.

Significantly, these mechanosensory inputs might combine with pathways mediated by other TRP and non-TRP pathways to provide multimodal integration at the odontoblast-afferent junction. Odontoblasts may function as specialized sensory intermediaries by detecting external stimuli, releasing mediators such as ATP and glutamate, and modulating pulpal afferents through purinergic and glutamatergic receptors. Our understanding of inflammation sensitization, pain transmission, and tooth pulp homeostasis is further enhanced by this non-TRP channel signaling. These tantalizing and fascinating hypotheses concerning the roles of odontoblasts in tooth pain need to be further determined through more selective manipulations of candidate molecular pathways in odontoblasts in vivo.

### 5.3. Roles of Dental Pulp Stem Cells in Pain

Dental pulp stem cells (DPSCs), which reside in proximity to the pulp’s vascular and neural components, offer more than just the ability to regenerate. DPSCs may also have the inherent ability to communicate with peripheral afferents because they are neural crest-derived cells. Their secretory activity could directly affect the behavior of sensory nerves and shape the local microenvironment. When DPSCs are stimulated by CGRP, they produce chemokines such as CXCL1 and CXCL8 [284]. Trigeminal neuronal excitability is increased by conditioned media from CGRP-primed DPSCs via CXCR2 signaling, demonstrating a feedback loop that intensifies neurogenic inflammation and nociceptor sensitization [284]. Therefore, pulp cells function as both modulators of afferent excitability and responders to nerve activity.

Dental pulp cells have context-dependent effects on sensory function. Exposure of these cells to polyinosinic:polycytidylic acid, which evokes inflammatory responses, induces oxidative stress and releases proinflammatory mediators from the dental pulp cells. This, in turn, promotes nociceptive sensitization [285]. In contrast, upon exposure to LPS, DPSCs exhibit immunomodulatory qualities, controlling immune-cell phenotypes and secreting anti-inflammatory cytokines through modulation by extracellular vesicles [286]. Their ability to resolve inflammation and repair the dentin–pulp complex supports the long-term restoration of pulp homeostasis by lowering prolonged nociceptor activation [286].

Overall, these findings indicate that dental pulp cells, especially DPSCs, might act as bridges between the pulp’s immune and neural components. Their capacity to both amplify and alleviate afferent sensitization reveals a non-TRP channel mechanism that regulates inflammation, pain, and pulp homeostasis.

## 6. The Roles of Pulpal Afferents and TRP Channels in Tooth Pulp Homeostasis

**Figure 5:**
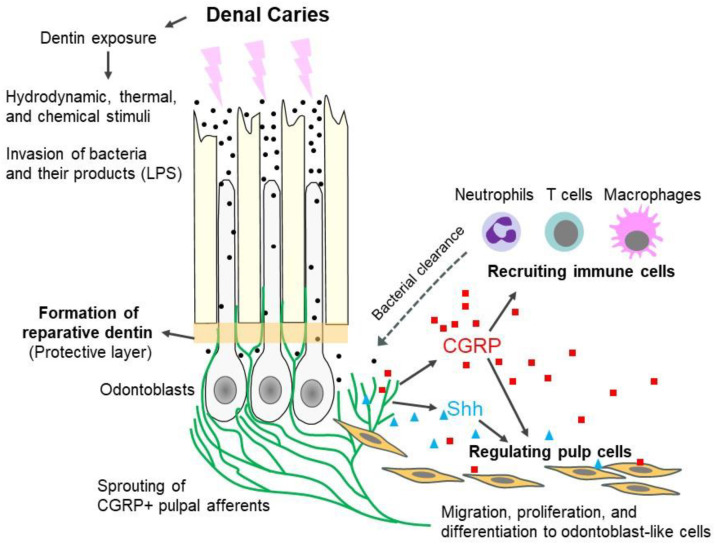
Pulpal afferents regulate host responses to and defense against tooth injury. CGRP, Calcitonin-gene-related peptide; Shh, Sonic hedge hog.

Sensory nerves play critical roles in maintaining homeostasis of innervating tissues, such as bone, skin, and the lungs [287,288]. TRPV1+ nerves regulate host responses in alveolar bone under periodontal infection or orthodontic tooth movement [6,289,290]. Although mice lacking TrkA, a receptor for nerve growth factor, do not have sensory innervation in the dental pulp [291], their tooth development appears to be normal, indicating that pulpal afferents are dispensable for normal tooth development. However, aside from sensory responses and nociception, pulpal afferents play important roles in maintaining tooth pulp homeostasis by regulating dental pulp cells and host responses under physiological and pathological conditions (Figure 5).

### 6.1. Pulpal Afferents Regulate Host Responses upon Injury

In human teeth, dental caries induce reactionary dentinogenesis, which is accompanied by the sprouting of nerve terminals within the reactionary dentin matrix and an increase in dendritic cells under carious lesions [292]. This suggests that neuro-immune interactions contribute to the protection and healing of tooth pulp. To determine the roles of sensory nerves in tooth pulp homeostasis, sensory nerve axotomy has been adopted. In rats, resection of the inferior alveolar nerve induces almost complete denervation of the mandibular molars without affecting the number of immune cells within the heathy (uninjured) dental pulp [293]. In teeth with dentin exposure, the number of immune cells (neutrophils, T cells, and macrophages) in pulp under injured dentin is increased [294]. This is accompanied by the sprouting of substance P+ or CGRP+ sensory nerve fibers in the pulp under the injured dentin [126,294,295,296]. Inferior alveolar nerve denervation reduces the recruitment of immune cells in injured teeth [294], indicating that sensory nerves play a role in regulating the host’s responses to tooth injury. In fact, sensory denervation profoundly regulates the responses of the tooth pulp to exposure injury [297]. Compared to innervated molars, inferior alveolar nerve denervation reduces the formation of a dense immune cell infiltration area within pulp under an injured site with an increased formation of necrotic tissues and periapical lesions [297]. Although nerve denervation involves all types of sensory afferents, peptidergic afferents might also play a role. Dentin injury in mouse molars increases the density of CGRP+ nerve fibers in pulp under exposed dentin, which, along with increased blood vessels, precedes the formation of reparative dentin [298]. Inferior alveolar nerve axotomy reduces angiogenesis and reparative dentin formation, whereas sympathetic denervation does not affect it. Olcegepant (BIBN-4096; a CGRP receptor antagonist) treatment also reduces angiogenesis and reparative dentin formation [298].

Peripheral neuropathy alters the activity of sensory afferents. Chemotherapy using paclitaxel induces peripheral neuropathy and persistent pain. In mice, paclitaxel treatment causes reduced pulpal CGRP-expressing afferents without changing neurofilament or PGP9.5-expressing afferents. In addition, tooth-injury-induced tertiary dentin formation is reduced in paclitaxel-treated mice without affecting odontoblast apoptosis [299], while inferior alveolar nerve chronic constriction injury induces long-lasting pain in rats. Moreover, rats with nerve injuries increase dentin-like calcified masses in molar pulp chambers, which are accompanied by a thick layer of predentin and increased CGRP+ nerves [300]. These studies suggest that pulpal afferents, likely CGRP+ peptidergic ones, contribute to those host responses to pulp injury that maintain pulpal homeostasis.

The role of CGRP in tooth pulp homeostasis has been further studied using *Calca* knockout mice [200]. CGRP induces the recruitment of neutrophils and monocytes after tooth injury. Since the depletion of neutrophils and monocytes decreases bacterial clearance from the tooth pulp and decreases nerve terminal density, neurogenic CGRP modulates the innate immune system’s ability to protect pulp from infection and tissue destruction.

### 6.2. Regulation of Dental Pulp Cells and Dental Pulp Stem Cells by Pulpal Afferents

Long-term observations of rats (up to 56 weeks after inferior alveolar nerve resection) have uncovered morphological and histological changes in the tooth pulp [301]. Denervation accelerated narrowing of the pulp chamber, increased the levels of senescence markers in pulp tissues, and increased the deposition of extracellular matrix and mineralized tissues. Dental pulp cells cultured from denervated teeth show decelerated cell proliferation, cell cycle arrest, and imbalanced matrix synthesis and degradation [301]. Denervation also regulates DPSCs under conditions of tooth injury [302]. Inferior alveolar nerve denervation in rats reduces organized tertiary dentin formation under the tooth injury area but increases ectopic mineralization in the pulp.

CGRP and the sonic hedgehog ligand are released from trigeminal nociceptive sensory neurons and increase the proliferation and odontogenic differentiation of dental pulp cells, suggesting their role in dentin formation [303]. The application of CGRP to the site of pulp exposure in vivo reduces ectopic calcification in denervated pulp, whereas BIBN4096, the CGRP antagonist, increases ectopic calcification in control mice. Moreover, the application of CGRP increases the migration of DPSCs (Stro-1+ or CD146+) to the area underneath the injured dentin in denervated molars, whereas BIBN4096 reduces migration to the injured site in control molars. DPSCs cultured from denervated molars exhibit greater mineralization capability but reduced migratory function in vitro. Applications of CGRP to a culture rescue the impaired migration of the DPSCs from denervated molars, supporting the idea that CGRP regulates DPSC migration [302].

### 6.3. Potential Contributions of TRP Channels in Pulp Homeostasis

Tooth injury increases sprouting of peptidergic nerve terminals under an injury site, and CGRP regulates pulpal immune responses and DPSC migration to the injury site, which protects the tooth pulp from injury. Since some TRP channels are highly expressed in peptidergic pulpal afferents and regulate neuropeptide secretion from pulp tissues [131], the environmental changes after tooth injury likely regulate TRP channel activities at the pulpal afferent terminals to modulate neuropeptide release and contribute to pulp and dental tissue repair. However, the roles of neuronal TRP channels in pulp homeostasis and dental tissue repair are not well known. In humans, TRPV1-like immunoreactivity in pulpal afferent terminals is greater in hypomineralized teeth than sound teeth [304], suggesting the potential roles of neural TRPV1 in dental tissue homeostasis.

Although TRPV1 is suggested to be expressed in non-neuronal cells, such as odontoblasts, its role in regulating dentinogenesis of odontoblasts is not clear. TRPV1 is also reportedly expressed in a human dental pulp fibroblastic cell line PF10, while capsaicin induces IL-6 expression and phosphorylation of p38 MAPK [305]. In the rat dental pulp cell RPC-C2A, capsaicin increases cell migration and alkaline phosphatase activity, suggesting a role for TRPV1 in the differentiation of dental pulp cells to odontoblast-like cells.

Although these data may indicate a potential role of neuronal and non-neuronal TRPV1 in dentinogenesis following tooth injury, more studies are necessary to establish the role of TRP channels in pulp homeostasis.

In primary human dental pulp cells, TRPA1 is upregulated under inflammatory conditions [285]. TRPA1 antagonists or knockdowns decrease the oxidative damage of dental pulp cells, suggesting that TRPA1 is a potential target for reducing inflammatory pulp damage [285]. The presence of TRPC1 and TRPC6 mRNA has been reported in acutely isolated rat and human odontoblasts, where they may function as mechanoreceptors [231]. In human DPSCs, knockdowns of TRPC1 [306] or TRPC6 [307] attenuated the process of odontoblast-like differentiation. These observations indicate that TRPC1 and/or TRPC6 may represent therapeutic targets in regenerative endodontics.

Non-TRP channels in odontoblasts are known to regulate dentinogenesis. Pharmacological activation of Piezo1 in human odontoblast cultures reduces mineralization, whereas knockdowns of Piezo1 enhance it, suggesting that Piezo1 contributes to pulp homeostasis [308].

## 7. Therapeutic Opportunities

Mechanistic studies on tooth pain largely rely on preclinical rodent models to draw inferences about pulp biology and TRP channel functions in humans. Although animal studies provide valuable mechanistic insights, there may be important interspecies differences in the anatomy, physiology, and pathogenesis. Such differences could shape neuronal and nonneuronal TRP channels’ contributions to pain signaling and pulp homeostasis across species. For this reason, caution is needed when extrapolating findings from animals to human dental pathology and further effort to integrate the human data with preclinical studies is necessary in future studies. Nonetheless, in this section, we may discuss potential therapeutic opportunities identified from preclinical mechanistic studies.

Toothache is severe and debilitating. Systemic antibiotics are ineffective in symptomatic irreversible pulpitis; therefore, operative intervention is mandatory. However, tooth-preserving root canal treatment is not an option in developing countries or economically disadvantaged populations. Therefore, caries prevention is of utmost importance. Capsaicin has a well-documented antibacterial effect (reviewed in [309]), independent of its action on TRPV1. Capsaicin was shown to inhibit the growth of *Streptococcus mutans*, a key contributor to cariogenic biofilm production [310]. In this study, the minimum inhibitory concentration (MIC) of capsaicin ranged from 1.25 to 5.0 µg/mL. A different study reported a higher MIC, 50 µg/mL [311]. By comparison, the degree of hotness traditionally accepted in the course of one meal equates to a consumption of 300 mg capsaicin by an adult weighing 60 kg (BfR Opinion No. 053/2011. www.bfr.bund.de; accessed on 6 October 2025). If 300 mg capsaicin is eaten in stew of 300 mL, it would translate into a concentration of 1 mg/mL capsaicin, much higher than the reported MIC against cariogenic bacteria. On the flip side, capsaicin may stimulate salivation [312] that may damage enamel. Notably, garlic (*Allium sativum*) is a traditional home remedy for toothaches. The TRPA1 agonist, allicin (the active ingredient in garlic), has proven bactericide activity against oral bacteria [313]. Thus, combining capsaicin with allicin may result in an even more efficient toothpaste. Although capsaicin and allicin each exhibit promising bioactive properties, their combined application in toothpaste formulations that support synergistic action, formulation stability, or clinical applicability requires future validation.

Beyond antimicrobial actions, TRP ligands also influence neurogenic inflammation. Infiltrating the inferior dental nerve with a 1% capsaicin solution reduced substance P expression in the rat dental pulp [314]. This observation provides a possible intervention for controlling pulpal neurogenic inflammation to protect pulp vitality. Capsaicin initially excites nociceptors and produces a burning sensation, but sustained or high-dose exposure results in functional desensitization of TRPV1-positive fibers [315]. This mechanism underlies its application in topical analgesics and experimental nerve models; nevertheless, direct intraoral use is impractical because of the severe pain upon administration and risk of mucosal irritation.

As mentioned above, oxidized linoleic acid metabolites generated by cytochrome P450 during pulpitis may function as endogenous TRPV1 agonists [215], contributing to inflammatory dental pain [316]. If this hypothesis holds true, *per os* TRPV1 antagonists may relieve inflammatory dental pain. As mentioned above, TRPV1 antagonists blocked acute pain induced by molar extraction [122]. However, the clinical development of these existing drugs may be repurposed to manage the chronic pain of pulpitis. In a Phase-2 clinical study, local capsaicin injection (into the buccal mucosa) was tried to control pain caused by wisdom tooth extraction (https://clinicaltrials.gov/study/NCT00088686; accessed on 6 October 2025). The results of this trial were not made public. However, the clinical development of first-generation TRPV1 antagonist compounds was discontinued due to hyperthermia and impaired thermoregulation. Although newer modality-selective and peripherally restricted agents may lessen these risks, their efficacy in dental pain remains unproven.

Studies on cold-induced tooth pain suggest a potential target for TRP modulation. Given the recent findings [192], TRPC5 can be targeted for treating cold hypersensitivity in teeth. The TRPC4/C5 antagonist, BI-1358894 (Boehringer Ingelheim, Ingelheim am Rhein, Germany), was well-tolerated in human volunteers and reduced the severity of experimental panic attack [317]. In a Phase-2, multicenter, randomized clinical trial, BI-1358894 showed antidepressive efficacy in patients refractory to standard treatment protocols (https://clinicaltrials.gov/study/NCT04521478; accessed on 10 October 2025). It would be interesting to determine whether BI-1358894 or another TRPC5 antagonist could alleviate cold-induced dental pain. However, because BI-1358894 acts on the CNS, its applicability to dental pain remains speculative. No preclinical or clinical data currently supports its effectiveness in odontogenic pain, and potential CNS-related adverse effects would need careful evaluation.

TRP channels also likely modulate pulp repair and regeneration. The silencing of TRPC1 or TRPC6 in human DPSCs markedly reduces their ability to differentiate into odontoblast-like cells. This finding highlights the role of these channels in regenerative endodontics, where the precise differentiation of pulp stem cells is crucial for the formation of reparative dentin. Therefore, pharmacological targeting of TRPC1 and TRPC6 could serve as a promising therapeutic approach to promote pulp repair and dentin formation, while also adjusting odontoblast mechanosensitivity to mitigate heightened pain signaling in inflamed pulp tissues [231,306,317].

Taken together, while TRP channels present promising molecular targets for antimicrobial, analgesic, and regenerative strategies in endodontics, their clinical application remains largely experimental. Rigorous evaluations of safety, underlying mechanisms, and translational feasibility are essential before these concepts can progress toward practical implementation.

## 8. Conclusions

Dental pulp is a complex sensory and immune organ. Pulpal afferents, odontoblasts, and immune cells form a single functional complex where TRP channels play a key role. Pulpal sensory afferents show distinctive neurochemical profiles predominantly innervation by myelinated TG neurons co-expressing markers of nociceptors. Tooth pulp afferents contribute to maintaining pulp homeostasis and coordinating reactions to infection by sensing danger signals and transducing potentially noxious stimuli. Moreover, they intimately coordinate their functions through interactions with non-neuronal cells in the tooth pulp, such as dental pulp cells, odontoblasts, and immune cells. TRP channels play critical roles in sensations and interactions that lead to protective sensory signaling while also amplifying pain during tooth pulp pathology. Evidence supports a neuro-immune-odontoblast axis, where TRP channel activity links molecular nociception to inflammation and repair mechanisms. This highlights the dual roles of neuropeptides such as CGRP and supports the view that odontoblasts serve active sensory and immune roles in the pulp. These intricates mechanisms need to be further validated in experimental animals by using more sophisticated genetic, chemogenetic, or optogenetic manipulations.

Critical questions still surround the dynamic modulations of TRP channels’ activity during the transition from acute to chronic pain, as well as the interactions of particular TRP subtypes within afferent populations. Filling these knowledge gaps is necessary to translate discoveries into precise clinical treatments. Ultimately, TRP channels have a strong therapeutic potential that positions them as the gatekeepers of immune and sensory functions for novel strategies aiming to reduce pain and maintain pulp vitality. Future studies that incorporate multi-omics approaches in human tissues, organotypic cultures, or advanced in vitro systems will be essential to improve translation and confirm the relevance of these mechanisms in human pulp biology.

## Data Availability

No new data were created or analyzed in this study. Data sharing is not applicable to this article.
